# Immunogenicity of a novel tetravalent dengue envelope protein domain III-based antigen in mice

**DOI:** 10.17179/excli2018-1664

**Published:** 2018-11-05

**Authors:** Hossein Fahimi, Majid Sadeghizadeh, Zuhair M. Hassan, Heidi Auerswald, Michael Schreiber

**Affiliations:** 1 Department of Genetics, Faculty of Biological Sciences, Tarbiat Modares University, Tehran, Iran; 2 Department of Immunology, Faculty of Medical Sciences, Tarbiat Modares University, Tehran, Iran; 3 Department of Virology, Bernhard Nocht Institute for Tropical Medicine, Hamburg, Germany

**Keywords:** dengue, serotypes, domain III, vaccine, tetravalent

## Abstract

Dengue virus is a mosquito-borne pathogen that causes dengue diseases. All four serotypes of dengue virus are infectious for humans. Therefore, an efficacious dengue vaccine should be tetravalent to provide protection against all types of virus. The goal of this study was to design a new tetravalent recombinant protein from envelope protein of dengue viruses to induce virus-neutralizing antibodies against all four serotypes in mice. A chimeric protein was designed from domain III of envelope protein of all serotypes of dengue virus. Four domain III fragments were linked together by alpha helix making linkers. The final sequence of the designed protein was analyzed *in silico* and the coding gene sequence was deduced by reverse translation. After cloning and expression of the recombinant protein (ED3-tetravalent protein), identity of the purified protein was confirmed using a pan-dengue specific monoclonal antibody in Western blotting. Then, the immunogenicity of the purified protein was studied in mice using antibody titration. The efficacy of induced antibodies in neutralization of the virus was studies by FRNT method. Furthermore, the induction of cellular immunity was studied by measurement of cytokines using ELISA method and measurement of lymphocyte proliferation using MTT assay. The ED3-tetravalent protein was able to enhance neutralizing immunogenic response against all four dengue serotypes; in similar way to that of tetravalent formulation of four individual domain III-based polypeptides. It is suggested that the ED3-tetravalent fusion protein can induce broadly neutralizing antibody responses against all four serotypes of dengue virus in mice.

## Introduction

Dengue virus is a mosquito-borne pathogen that causes dengue fever, dengue shock syndrome (DSS), and dengue hemorrhagic fever (DHF). Recently the reports for dengue infections have been increased, especially among people who live in warm and humid areas. These viruses are transmitted to humans through the bites of "*Stegomyia”*; especially *Aedes albopictus* and* Aedes aegypti* (Gubler, 1998[17], 2002[18]; Whitehorn, 2012[39]). All dengue serotypes (serotypes 1-4) can infect human and because there is no efficient cross protection between the various serotypes, a perfect dengue vaccine should be a tetravalent vaccine (Guzman et al., 2010[20]). According to the report from “WHO” website, Sanofi Pasteur developed a live-attenuated multivalent vaccine, Dengvaxia (CYD-TDV) which was recently licensed in Mexico and several countries. However, considering some safety issues, designing a novel tetravalent subunit vaccine with efficient immune-protective properties is remained as an attractive subject. 

Several publications have reported the creation of effective vaccine candidates on the basis of conventional live attenuated viruses, inactivated viruses, from infectious clone derived attenuated viruses, and genetic vaccines (Swaminathan and Khanna, 2010[37]; Murrell et al., 2011[31]; Schmitz et al., 2011[33]). Recently, considerable research has also been directed towards the production of recombinant subunit vaccines (Whitehead and Subbarao, 2017[38]). Since subunit vaccines use merely a specific portion of pathogen, vaccines produced this way can be considered easier, cheaper, safer and more stable than the live attenuated dengue vaccines (Clements et al., 2010[8]). Therefore, most of recent investigations are focused on envelope protein of dengue virus (E protein). The E protein mediates virus entry into host cells via receptor-binding (Henchal and Putnak, 1990[21]). The dengue E protein consists of three domains (I, II, and III) (Modis et al., 2004[28]) of which the domain III (ED3) appears to play critical roles in the next step of virus entry into the host cell. It is also very potent in induction of immune-protective responses against the virus. It has been reported that the anti-ED3 specific monoclonal antibodies can block virus entry and infectivity. In contrast, domains I or II-specific antibodies have represented lower avidity and cross neutralization properties (Chávez et al., 2010[5]; Modis et al., 2005[29]). Several investigations have shown that the recombinant ED3 proteins can inhibit dengue infectivity, and induce dengue-neutralizing immunoglobulin in mice*. *Taken together, the critical region of the E protein for vaccine design and development have been identified on ED3 (Hermida et al., 2006[22]; Mota et al., 2005[30]; Babu et al., 2008[1]; Leng et al., 2009[27]; Khanam et al., 2006[23]; Zhang et al., 2015[41]; Chiang et al., 2013[7]; Suzarte et al., 2014[36]; Fahimi et al., 2013[15], 2014[12], 2018[13]; Kulkarni et al., 2017[24]; Gottschamel et al., 2016[16]). In this study, a new tetravalent protein was designed and expressed based on consensus ED3 of all dengue serotypes. The designed chimeric protein (ED3-tetravalent protein) was expressed in bacterial host and after purification and formulation with proper adjuvant the analysis of the immunogenic responses was carried out on mice. It was shown that this fusion protein can induce antibodies in mice which were able to cross-neutralize all dengue serotypes *in vitro. *This approach can be very effective for simultaneously induction of protective antibodies for all dengue virus serotypes by a recombinant protein, and potentially offers advantages over the tetravalent mixture of four domain III fragments. 

## Material and Methods

### Cells and viruses

Four serotypes of the dengue virus, (DENV 1 strain 16007, DENV2 strain Th-NH-81/93, DENV3 strain BR/D3LIMHO/ 2006, DENV4 strain PH/BID-V3361/1956), were used for this study. Virus propagation and titration was performed in Vero B4 cells. All obtained from Bernhard Nocht Institute for Tropical Medicine (BNITM, Germany). Cells were maintained in Dulbecco's Modified Eagle's Medium (DMEM) with 4.5 g/l glucose [were obtained from PAA Laboratories, Colbe, Germany] supplemented with 10 % (v/v) heat inactivated fetal calf serum (FCS) [PAA], 2 mM glutamate [PAA], 50 U/ml penicillin and 50 µg/ml streptomycin [Gibco] in a 5 % CO_2_ humidified incubator, at 37 °C. 

### Designing and construction of ED3 sequences

Four consensus ED3 sequences were provided corresponding to the four dengue serotypes and designed a tetravalent antigen by fusing these fragments together, as reported previously (Fahimi et al., 2016[14]). Briefly, the amino acid sequences of ED3 from all four serotypes were obtained from GenBank and multiple sequence alignment by using ClustalW software was performed in order to identify an amino acid sequence common in different isolates of the four serotypes. Four consensus ED3 peptide sequences were used for designing a tetravalent fusion protein (ED3F: GenBank accession no. JN985899). In order to achieve high expression level, the codons were optimized for *Escherichia coli* by using Optimizer (http://genomes.urv.es/OPTIMIZER/). For cloning purposes, restriction sites of *Nde* I and *Xho* I enzymes were introduced at the 5' and the 3' sites, respectively. The target gene was synthesized by Eurofins MWG Operon (Germany), and sub-cloned into pET21a(+) expression vector (Novagen). As a result, the carboxyl terminus of the recombinant protein contains a hexa-histidine tag (His_6_-Tag). 

### Expression and purification of recombinant protein

The constructed expression vector (pETD3F) was transformed into *E. coli* DH5α for plasmid amplification and into *E. coli* Origami(DE3) for protein expression. A single colony of transformed *E. coli* was grown overnight at 37 ºC in 5 ml LB medium containing 50 μg/ml ampicillin, 12.5 μg/ml tetracycline, and 15 μg/ml kanamycin (Sigma-Aldrich, St. Louis, MO, USA). Then the cultures were diluted 1:100 in LB medium containing antibiotics as described before and further incubated at 37 ºC. The cultures were induced in the logarithmic phase (at OD_600_ of 0.6) by addition of isopropyl β-D-1-thiogalactopyranoside (IPTG) to a final concentration of 1 mM. After 4 hours, the expression of the recombinant ED3F was analyzed by SDS-PAGE (Laemmli, 1970[25]). The recombinant protein prepared from soluble fraction of Origami(DE3) cell lysate were purified using Nickel-nitrilotriacetic acid (Ni-NTA) (Qiagen, Germany) resin under native condition, according to manufacturer's instruction. The protein concentrations were analyzed by Bradford protein assay (Bradford, 1976[4]). Furthermore, the four consensus ED3 proteins were expressed and assessed for immunogenicity, similar to the previous report (Fahimi et al., 2014[12]). The origami (DE3) strain of *E. coli* was used as an expression host. 

### Immunoassay procedures

For Western blot analysis the purified proteins were applied on a 10 % SDS-PAGE and transferred onto nitrocellulose membrane by using a semidry (Biorad, California, USA) system (Tris 12.5 mM, Glycine 96 mM, SDS 0.03 %, and methanol 10 % with pH 8.3). The membrane was incubated in the blocking buffer of 5 % skimmed milk at 4 ºC overnight. It was then incubated in a 1:1000 diluted primary antibody, anti-HisTag (Abcam, UK) or pan-dengue specific monoclonal antibody (cat no. MAB4043, Abnova), with gentle shaking for 2 hours at room temperature (RT). The membrane was washed three times with PBST (PBS containing 0.1 % Tween 20) and then incubated in secondary antibody, a 1:2000 dilution of HRP-conjugated rabbit anti mouse IgG antibody (Abcam, UK), with gentle shaking for 1 h at RT. After a 15 min washing with PBST, the detection was performed using diaminobenzidine (DAB) as a chromogenic substrate. Dot blotting analysis was performed using a very similar method like Western blotting. Briefly, recombinant proteins were dotted on nitrocellulose membrane strips and were reacted with diluted serum samples from the immunized animals. Blocking, antibody incubation, and detection methods were identical with Western blotting, except the serum dilutions were 1:10 and the chromogenic substrate was 4-Chloro-1-naphthol (Sigma-Aldrich, St. Louis, MO, USA). 

### Animal immunizations

Male BALB/c mice were obtained from Pasteur Institute (Tehran, Iran). Groups of six mice (6-8 weeks of age) were immunized subcutaneously with the recombinant proteins formulated with Freund's complete adjuvant (FCA, Sigma-Aldrich, St. Louis, MO, USA) for priming (day 0), and with Freund's incomplete adjuvant (FIA, Sigma-Aldrich, St. Louis, MO, USA) for two booster immunizations (days 14 and 28). A group of mice were injected with PBS in combination with adjuvant, as negative control group (Table 1(Tab. 1)). Since immunogenicity properties of FCA/ FIA are more potent than popular adjuvant-alum, FCA/FIA was used for this experiment. The mice were sacrificed and blood collected, 14 days after the last immunization, and pooled sera were stored at -80 ºC until use. All procedures were performed according to the guidelines of the Medical Ethics Committee of Tarbiat Modares University, Tehran, Iran (D52/6854).

### Measurement of humoral antibodyresponse in immunized mice

The levels of anti-ED3(1-4)/anti-ED3F IgG in the serum samples were determined by using an indirect enzyme-linked immunosorbent assay (ELISA). Polystyrene 96-well plates (Nunc-Immuno Plate MaxiSorp surface, Nunc, Denmark) were coated with purified proteins (0.1 µg/well) then were incubated overnight at 4 °C. The washing step was performed three times with PBST (PBS-buffer containing 0.05 % Tween 20) and the blocking step was carried out by using 5 % skimmed milk in PBS for 1 h at 37 ºC. Mouse serum samples were serially diluted (100 μl/well) in blocking buffer (1:100 to 1:12800, and incubated for 2 h at 37 ºC. After washing, bounded IgG was detected with horseradish peroxidase-conjugated rabbit anti-mouse IgG Fc (Abcam, UK), diluted 1:4000 in blocking buffer. Finally, 100 μl of 3,3',5,5'-tetramethylbenzidine (TMB) as chromogenic substrate were added and the plates were incubated at RT for 15 min. The reaction was stopped by adding 100 μl of 2N sulfuric acid and the absorbance at 450 nm was performed by using ELISA reader (Bio-Rad).

### Focus reduction neutralization tests (FRNT)

Anti-dengue neutralizing antibodies were detected by using a focus reduction neutralization test (FRNT), as reported previously (Leng et al., 2009[27]). Briefly, the pooled serum samples from each group were heat inactivated for 30 min at 56 °C. Vero B4 cells were seeded into 96-well cell culture plates [Sarstedt] to reach a confluence of 90 % after 24 h. The pooled sera were used to prepare twofold serial dilutions in DMEM medium starting at 1:10. Then, the diluted sera were mixed 1:1 with virus suspensions in 96 well cell culture plates and incubated for 1 h at 37°C. Virus suspensions were contained ~100 focus forming unit (FFU) of each serotype per well. In order to viral adsorption, the Vero cell cultures were inoculated with the prepared serum-virus mixes and incubated for 1 h. Remaining infectivity was then assayed on cell monolayers overlaid with DMEM containing 0.8 % (w/v) methylcellulose with 20 mM Tris. After 2 days of incubation at 37 °C with 5 % CO_2_, the overlay medium was removed from the wells, and the Vero cells were washed with PBS. The cells were fixed for 20 min in 3.7 % formaldehyde/PBS and were washed with PBS. Then the cells were permeabilized with 0.5 % triton X-100/PBS for 20 min and blocked with 10 % fetal calf serum/PBS for 30 min. Infected cells were detected by DENV serotype-specific monoclonal antibodies. After washing with PBS, antibody-labeled cells were detected using a HRP-conjugated secondary antibody [Biorad, California, USA]. The labeling was visualized using precipitating tetramethylbenzidine (TMB) substrate [Mikrogen Diagnostik, Neuried, Germany]. The highest serum dilution that reduced the number of focuses by at least 50 % (FRNT_50_) in comparing with the control was calculated as the end-point neutralizing antibody titer. The control samples were containing the virus and mock-immunized sera as well as samples with virus alone.

To investigate reactivity of the serum samples with dengue infected cells a very similar method with FRNT was used. Briefly, Vero B4 cells were seeded into 96-well cell culture plates to reach a confluence of 90 % after 24 h. The infection step was performed by adding of 100 tissue culture infective dose 50 (TCID50) of each serotype of dengue virus. Cells were then overlayed (at 37 °C with 5 % CO_2_ for 48 h). Afterward, the overlay medium was removed, and the cells were washed with PBS. Cells were then permeabilized and blocked according to the mentioned procedures. Infected cells were detected by 1:10 dilution of different serum samples from the immunized mice for 2 h. After washing with PBS, the serum-labeled cells were detected using a HRP-conjugated secondary antibody. The labeling was visualized using TMB substrate and detected by light microscope. 

### Lymphocyte proliferation assay

We used a previously reported MTT assay procedure for lymphocyte proliferation assay (Babu et al., 2008[1]). Briefly, splenocytes were cultured in 96-well cell culture plates (Nunc, Denmark) and the aliquots of the antigens were then dispensed into the wells, at a final concentration of 10 μg/ml of antigen. Wells with splenocytes from mock-immunized mice were included as negative control. As well as ConA-stimulated cell suspensions were also included as positive control (ConA: Sigma-Aldrich, St. Louis, MO, USA). Cultures were incubated at 37 ºC in 5 % CO_2_ humidified incubator for 1-3 days. The MTT proliferation assay was performed using 3-(4,5-dimethylthiazol-2-yl)-2,5-diphenyl tetrazolium bromide. After incubation, cells were treated with MTT solvent for 15 minutes at RT. Absorbance was measured at OD = 570 nm.

### Cytokine assay

To analyze the phenotype of the specific cellular immune response induced by the recombinant protein, the supernatant from each triplicate well from splenocytes cultures were pooled after 72 h and were frozen at -80 ºC until use. To examine the level of the Th1-type cytokine of INF-γ and Th2-type cytokine of IL-4, commercially cytokine quantitative ELISA kits (mouse INF-γ and IL-4 kits, Qiagen, USA) were used according to the manufacturer's instructions. 

### Statistical analysis

The data were analyzed using one-way ANOVA of SPSS software version 22 and were expressed as mean ± standard deviation (SD) (from certain independent experiments) and the P-values of <0.05 were considered statistically significant.

## Results

### Tetravalent antigen design and expression

**Figure 1:**
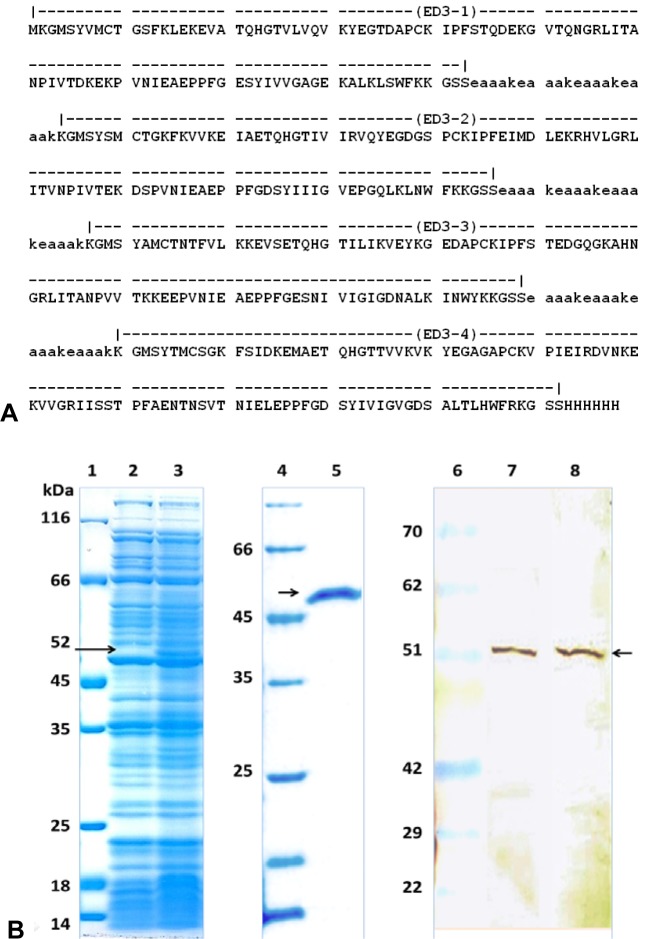
Amino acid sequence, purification and characterization of the ED3-F antigen. (A) Amino acid sequence of the tetravalent ED3-F antigen. The four ED3 sequences ED3-1, ED3-2, ED3-3 and ED3-4 were fused together by a repetitive linker sequence (lower case letters). The ED3-F coding sequence was cloned into the pET21a plasmid fused to a His_6_-Tag coding sequence. (B) Lanes 2 and 3, protein production profiles of bacterial lysates prepared before (lane 2) and after IPTG induction (lane 3). Soluble cell extracts were analyzed on a 10 % SDS polyacrylamide gel and stained with Coomassie Brilliant Blue. The arrow indicates the 52 kD band representing the ED3-F protein. Lane 5, purified ED3-F antigen after the second purification step. Lane 7 and 8, ED3-F tested for immune reactivity against anti-dengue monoclonal antibodies (lane 6) an anti-His_6_-Tag antibody (lane 7) by Western blot. Lanes 1, 4 and 6 protein molecular weight markers

Four consensus ED3 sequences of dengue serotypes were fused together by appropriate hydrophobic linkers as a tetravalent fusion protein (ED3F) (Figure 1A(Fig. 1)). The ED3F protein was predicted to be a 479 amino acid protein with molecular weight of~52 kDa. In this recombinant protein, the first amino acid residue is a vector-encoded methionine followed by 477 amino acid residues encompassing domain III of all four serotypes of dengue envelope protein, the linker sequences, and His_6_-Tag sequence. The designed pETD3F construct was transformed into Origami(DE3) host cells. It has been shown that the Origami strain of *E. coli* with glutathione reductase (*gor*) and thioredoxinreductase (*trx*B) mutations supports the formation of disulfide bridges in *E. coli *(Saejung et al., 2006[32]). To identify clones capable of expressing the desired recombinant protein, small-scale cultures of the positive clones (selected by PCR) were subjected to IPTG induction. A typical induction experiment comparing the polypeptide profile of un-induced and induced cultures is shown in Figure 1B(Fig. 1). It is evident that IPTG induction results in the expression of a protein corresponding to the expected molecular weight of ~52 kDa. The purified recombinant ED3F protein was characterized by Western blot analysis. It was shown that the fusion protein reacts specifically with pan-dengue specific antibody and anti-His-Tag monoclonal antibody (Figure 1B(Fig. 1)). Together, these results demonstrated the successful purification and characterization of the designed tetravalent fusion protein.

### Immunogenicity of recombinant proteins

In order to test the immunogenicity of the ED3F protein, a group of mice were immunized subcutaneously with ED3F protein three times at two-week intervals. In parallel, groups of mice were immunized with four monovalent and a tetravalent formulation of ED3 proteins (ED3 (1-4) mixture) by using the same method (Table 1(Tab. 1)). The mice in negative control (G7) were immunized with PBS in combination with adjuvant (mock-immunization).

**Figure 2:**
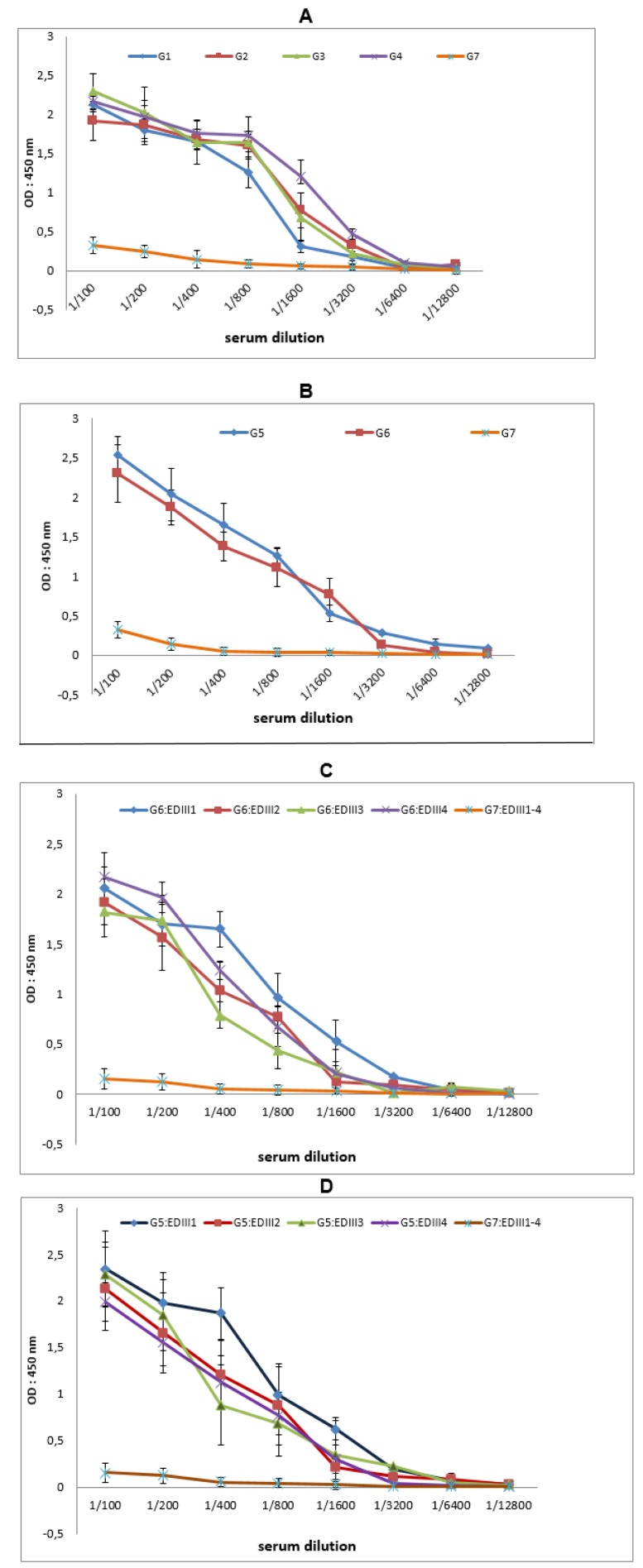
Antibody titers to ED3 in pooled sera from immunized mice. Sera collected from BALB/c mice (n=6) that had been immunized subcutaneously with the recombinant ED3 antigens were pooled for each group and the pooled sera were tested at various dilutions for their reactivity to ED3 antigens by ELISA. The mock-immunized mice serum pool G7 was used as a control. (A) The four ED3 proteins ED3-1, ED3-2, ED3-3 and ED3-4 were coated onto ELISA plate as individual antigens or as a mixture in order to determine anti-ED3 antibody titers in the pooled sera from monovalent immunized mice. (B) The ED3-F protein was coated onto ELISA plate as antigen to test the anti-ED3-F antibody response in pooled sera from ED3(1-4) (G5) and ED3-F (G6) immunized mice. The results showed that the mice in both G5 and G6 groups developed antibody responses towards the tetravalent ED3-F antigen. (C and D) The reactivity of the pooled sera from G6 and G5 to monovalent ED3 antigens was investigated, respectively. The monovalent ED3-1, ED3-2, ED3-3, and ED3-4 recombinant antigens were tested by ELISA as individual antigens. As a control, a pooled serum from G7 was tested against the ED3(1-4) antigen.

Sera from immunized mice were collected and analyzed for anti-ED3F and anti-ED3(1-4) antibodies by ELISA. The results shown in Figure 2(Fig. 2) (A and B) indicate that the mice developed high-titer antibody responses against the corresponding antigens. To investigate the profile of expected tetravalent response following immunization with ED3F protein, reactivity of anti-ED3F serum with four ED3 proteins was analyzed separately by using indirect ELISA (Figure 2C(Fig. 2)). As a comparison, reactivity of pooled serum from G5 with four ED3 proteins was also analyzed (Figure 2D(Fig. 2)). Interestingly, these data revealed that the potential of tetravalent fusion protein in developing high and balanced titer of tetravalent antibody response against all four ED3 types.

**Figure 3:**
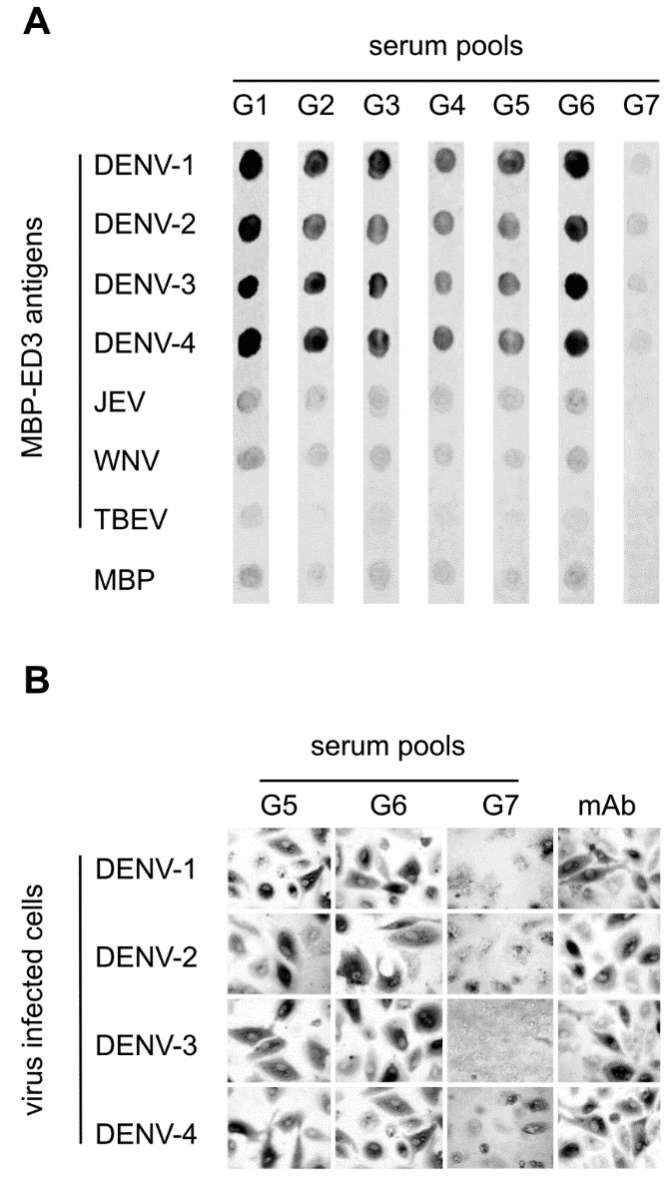
Reactivity of immune sera against recombinant ED3 and virus infected cells. (A) All pooled sera from immunized mice were tested for reactivity against recombinant ED3 antigens from DENV, JEV, WNV and TBEV by an immune-dot blotting assay. Recombinant antigen was immobilized on nitrocellulose strips, incubated with sera and bound antibodies were detected by a secondary HRP conjugated antibody and 4-CN staining. (B) Pooled sera from tetravalent immunizations (G5, G6) were tested for reactivity against virus antigen. Vero B4 cells were infected with DENV and cells were immobilized using formaldehyde and permeabilized with Tween20. Sera, 1:2 diluted were used as a primary antibody and a secondary HRP-conjugated anti-mouse IgG antibody was used for microscopic detection of the positively stained cells. A commercial pan-dengue specific monoclonal antibody diluted 1:10 dilution was used as a reference (Abnoova, Germany). These data prove the dengue vaccine immune sera can recognize the native dengue viruses in cell culture.

Furthermore, the specific reactivity of anti-ED3 serum samples with dengue and non-dengue flaviviral recombinant ED3 proteins were investigated. According to the results of dot blotting, all serum samples from the immunized mice groups reacted with dengue ED3 proteins, there was no significant reaction between the antisera and ED3 proteins of other non-dengue flaviviruses (JEV, WNV, and TBEV) (Figure 3A(Fig. 3)). All dengue antigens were reacted with undiluted serum samples from the immunized animals. The resulted cross-reactivity between the different serotypes can be due to the non-quantitative nature of the assay. The reactivity of tetravalent antisera (from G5 and G6) with dengue antigens also were investigated in cell culture (Figure 3B(Fig. 3)). All four serotypes of dengue virus were recognized by the tetravalent antisera, whereas dengue viruses were not detected in mock-infected cell. These data prove the dengue vaccine immune sera can recognize the native dengue viruses in cell culture. As additional control, serum pools and control MAb tested against uninfected cells that resulted in no reaction response. 

### Cross neutralization of all four dengue serotypes

The major objective of this study was to explore whether ED3F can induce cross-neutralizing antibodies. The neutralizing ability of the antiserum from the different immunized groups against the four serotypes of dengue virus was assessed using FRNT. As shown in Table 2(Tab. 2), anti-ED3F serum reduced the number of FFU when was pre-incubated with virus. These results indicate that tetravalent ED3F protein is able to elicit cross-neutralizing antibodies to block all four serotypes of dengue virus. While, all of the monovalent antiserum samples (sera G1-G4) showed cross-neutralizing effects, serotype specific neutralization was observed only in high diluted sera (1:80 and 1:40). Interestingly, in a very similar way with anti-ED3F serum, the serum from immunized mice with tetravalent formulation of four ED3 proteins (G5) cross-neutralized all four serotypes of dengue virus. Complete neutralization of dengue viruses by all anti-ED3 sera was observed up to a dilution of around 1:40. These data revealed the potential of recombinant envelope protein domain III in developing neutralizing immune responses against dengue virus. However, it should be noted that background neutralization was observed by high titers of serum from mock immunized group (G7).

### Cell proliferation and cytokine profiling assays

**Figure 4:**
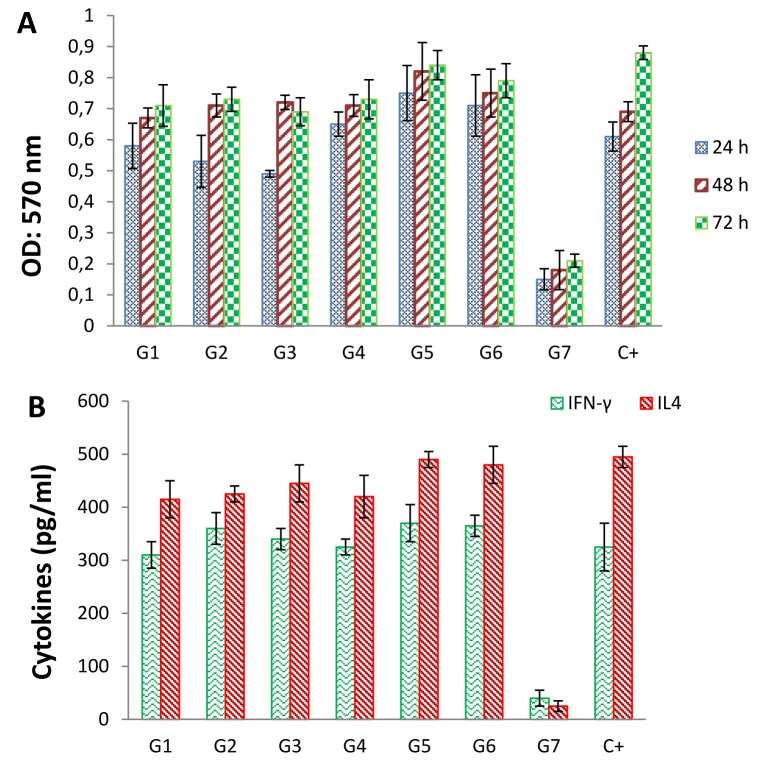
Lymphocyte proliferation and cytokine assays. (A) Splenocytes from immunized mice (G1-G6) were prepared 2 weeks after the last immunization, and re-stimulated with the corresponding antigens for 24, 48, and 72 h. This assay was done using a MTT cell proliferation assay. The splenocytes from mock-immunized group, stimulated by the tetravalent formulation of ED3s, was considered as negative control (G7). The splenocytes of the immunized mice showed high level of proliferation in comparison with the negative control. Splenocytes from unimmunized mice responded to ConA nonspecifically (C+). There is significance in cell proliferation between different time points (24 h and 72 h). In comparing with mock immunized group (G7), it can be concluded that the cell proliferations are higher in dengue vaccine immunized mice. (B) Cytokine assay upon *in vitro* re-stimulation of splenocytes. Splenocytes from mice (G1-G6) were re-stimulated by the corresponding antigens, and the level of two cytokines (INFγ and IL4) was measured using commercial quantitative ELISA kits. The splenocytes from mock-immunized mice were stimulated by the tetravalent ED3 mixture and were considered as negative control (G7). The splenocytes from non-immunized mice (G8) were stimulated by ConA *in vitro* as a positive control. Data are presented as the mean cytokine concentrations from triplicate wells.

According to the MTT assay in three days intervals, the splenocytes of the immunized mice showed high proliferation in comparison to the negative control (Figure 4(Fig. 4)). Splenocytes in negative control did not show comparable proliferation when stimulated with tetravalent formulation of ED3 proteins, indicating specificity of the proliferation. Splenocytes from G5 showed better proliferation than other groups. Splenocytes from unimmunized mice responded to stimulation by ConA, nonspecifically (as a positive control for monitoring of the stimulation level). Comparison among duration of treatments (24 h and 48 h) did not display negative effects or significant differences, although the overall trend of proliferation was upward. Though, there is significance in cell proliferation between different time points (24 h and 72 h). In comparing with mock immunized group (G7), it can be concluded that the cell proliferation is higher in dengue vaccine immunized mice. Furthermore, quantitative ELISAs were used to detect the secreted IFN-γ and IL-4 cytokines from splenocytes of the immunized mice re-stimulated with the corresponding antigens (Figure 4(Fig. 4)). Splenocytes of unimmunized mice stimulated by ConA were included in cytokine assay as a positive control group (G8). In comparison with the negative control group (G7), the concentration of two measured cytokines increased markedly in all ED3-immunized groups. However, in both MTT and cytokine assays no significant differences between the immunized groups were detected. It is likely due to application of high concentration of antigens. 

## Discussion

Because of an increased risk of developing DHF and DSS in dengue-infected individuals with a previous heterologous dengue infection (Gubler, 2002[18]), an effective dengue vaccine must provide solid and long-lasting cross-protection against all four serotypes. So, we attempted to develop a tetravalent dengue vaccine candidate by using the envelope protein domain III. Recombinant ED3 proteins show immunogenic and protective properties in animals (Crill and Roehrig, 2001[10], Yang et al., 2012[40], Coconi-Linares et al., 2013[9], Block et al., 2010[3]). Although ED3-based vaccine represents only a fraction of the envelope protein, the removal of other epitopes that elicit non-neutralizing cross-reactive antibodies, may reduce the risk of disease progression to DHF and DSS (Bernardo et al., 2009[2]). The immunogenicity of ED3 fragment of type 3 dengue virus was reported previously (Fahimi et al., 2014[12]). However, this study was aimed to design a tetravalent vaccine candidate in the form of a physical mixture of four ED3 fragments or in a single chimeric protein. One approach that has been suggested to minimize the possibility of antibody-dependent enhancement of infection, occurring after vaccination, is to design a tetravalent subunit vaccine containing potent neutralizing epitopes against all four serotypes. In the current study, a tetravalent ED3-based recombinant antigen (ED3F) was designed and expressed. To facilitate easy purification, ED3F protein and four ED3 proteins with a C-terminal 6x-HisTag were expressed in soluble form and without any bulky fusion partner like MBP or GST. Immunogenicity of this tetravalent fusion protein was verified and also compared with tetravalent formulation of ED3s. The reactivity of anti-ED3F antiserum against ED3 proteins demonstrated that this new tetravalent immunogen elicited antibody responses against all four ED3 types. The specific reactivity of anti-ED3 sera with recombinant dengue ED3 proteins was shown in a dot blotting assay, in comparing with ED3 proteins of the other flaviviruses. Furthermore, the tetravalent anti-ED3 serum samples were able to recognize all dengue serotypes in cell culture. On the other hand, the results of neutralization assays revealed that the elicited tetravalent immune response was capable to neutralize all four serotypes of dengue virus. These results indicated that the (EAAAK)_4_ linkers were effective in equal expression and supportive for accessibility of epitopes in ED3F structure. It was reported that a tetravalent dengue vaccine candidate with (Gly4Ser)3 linkers between ED3 fragments, results in induction of an unbalanced immune response in mice (Chen et al., 2007[6]). The authors discussed that some antigenic determinants may be buried in the protein interior. Hence, a more suitable linker was used in a way to balance four consensus ED3 proteins and expose antigenic determinants. In addition, cell-mediated immunity was evaluated by antigen-stimulated splenocyte proliferation assay and cytokine production *in vitro*. Splenocyte from all immunized mice showed significant levels of proliferation in response to *in vitro* re-stimulation with ED3 antigens. The results of cytokine assays showed significant rise in the levels of IFN-γ and IL-4. IgG2a and IgG3 responses are enhanced by IFN-γ, and B cells are capable of secreting IgG1 in the presence of IL-4 (Stevens et al., 1988[35]; Le Gros et al., 1990[26]). IFN-γ has been described as a mediator of the cellular immune response and plays a distinctive role in antiviral activity against dengue viruses (Shresta et al., 2004[34]; Dejnirattisai et al., 2008[11]). Furthermore, it has been shown that IFN-γ is associated with protection against dengue fever and/or viremia in human (Gunther et al., 2011[19]). Based on these data and the presence of IFN-γ as a marker of the Th1 response and IL-4 as a marker of the Th2 response, it may be suggested that both of the Th1- and Th2-type immune responses were induced following vaccination of mice. Although, the results of this study were not revealed significant differences between the application of a mixture of four monovalent antigens and a tetravalent antigen, but a single tetravalent antigen shows basic advantages over physically mixture of four monovalent antigens such as ease of production and formulation.

In conclusion the tetravalent ED3F fusion protein was able to enhance cross-neutralizing antibody responses against all four serotypes of dengue virus. The new ED3F antigen can be used to develop a tetravalent subunit vaccine candidate for prevention of dengue infections.

## Acknowledgement

Our grateful thanks are due to Prof. Bernhard Fleischer (from BNITM) for his valuable help, and also Kati Franzke (formerly from BNITM, now Federal Research Institute for Animal Health, Greifswald - Insel Riems, Germany) for technical collaborations. The recombinant MBP-ED3 fusion proteins from other flaviviruses were kindly provided by Dr. Kati Franzke (BNITM, Germany). Infected cells were detected by DENV serotype-specific monoclonal antibody which was kindly gifted by P. Buchy, Institute Pasteur Cambodia. 

## Conflict of interest

The authors declare that they have no conflict of interest.

## Figures and Tables

**Table 1:**
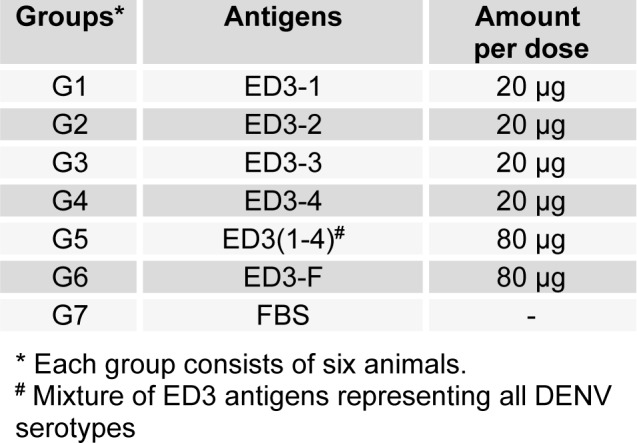
Antigens used for immunization of the mice

**Table 2:**
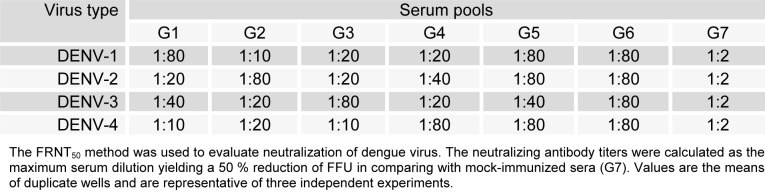
Virus-neutralization titers of serum pools from immunized mice
